# Improving accelerated 3D imaging in MRI-guided radiotherapy for prostate cancer using a deep learning method

**DOI:** 10.1186/s13014-023-02306-4

**Published:** 2023-07-01

**Authors:** Ji Zhu, Xinyuan Chen, Yuxiang Liu, Bining Yang, Ran Wei, Shirui Qin, Zhuanbo Yang, Zhihui Hu, Jianrong Dai, Kuo Men

**Affiliations:** 1 National Cancer Center, National Clinical Research Center for Cancer, Cancer Hospital, Chinese Academy of Medical Sciences and Peking Union Medical College, Beijing, 100021 China; 2 School of Physics and Technology, Wuhan University, Wuhan, 430072 China

**Keywords:** Accelerated 3D MRI scanning, High-quality MRI image, Cycle-consistent adversarial networks, Image quality, Deep learning, 1.5 T MR-linac

## Abstract

**Purpose:**

This study was to improve image quality for high-speed MR imaging using a deep learning method for online adaptive radiotherapy in prostate cancer. We then evaluated its benefits on image registration.

**Methods:**

Sixty pairs of 1.5 T MR images acquired with an MR-linac were enrolled. The data included low-speed, high-quality (LSHQ), and high-speed low-quality (HSLQ) MR images. We proposed a CycleGAN, which is based on the data augmentation technique, to learn the mapping between the HSLQ and LSHQ images and then generate synthetic LSHQ (synLSHQ) images from the HSLQ images. Five-fold cross-validation was employed to test the CycleGAN model. The normalized mean absolute error (nMAE), peak signal-to-noise ratio (PSNR), structural similarity index measurement (SSIM), and edge keeping index (EKI) were calculated to determine image quality. The Jacobian determinant value (JDV), Dice similarity coefficient (DSC), and mean distance to agreement (MDA) were used to analyze deformable registration.

**Results:**

Compared with the LSHQ, the proposed synLSHQ achieved comparable image quality and reduced imaging time by ~ 66%. Compared with the HSLQ, the synLSHQ had better image quality with improvement of 57%, 3.4%, 26.9%, and 3.6% for nMAE, SSIM, PSNR, and EKI, respectively. Furthermore, the synLSHQ enhanced registration accuracy with a superior mean JDV (6%) and preferable DSC and MDA values compared with HSLQ.

**Conclusion:**

The proposed method can generate high-quality images from high-speed scanning sequences. As a result, it shows potential to shorten the scan time while ensuring the accuracy of radiotherapy.

**Supplementary Information:**

The online version contains supplementary material available at 10.1186/s13014-023-02306-4.

## Introduction

MR-linac is an innovative linear accelerator integrated with a magnetic resonance imaging (MRI) system. It enables the use of online adaptive planning based on the daily patient anatomical changes during every fraction [1–4]. The imaging system of Unity (Elekta) can currently provide 1.5 T low-speed, high-quality (LSHQ 3D) and high-speed, low-quality (HSLQ 3D) sequences for daily adaptive planning and position-validation. The LSHQ sequence can provide high-quality daily MR images, but its scan time is long for adaptive radiotherapy. The scan time of the HSLQ sequence (about 117 s) is much shorter than that of LSHQ sequence (about 411 s); however, the inferior image quality does not provide sufficient anatomical detail for daily radiotherapy. High quality image can provide both the extent and position of a tumor precisely, so as to irradiate tumors with high accuracy. However, this time-consuming issue is a significant challenge for MRI-based adaptive radiotherapy. Long scan times mean that the patient is likely to become uncomfortable and move [5]. The case of spontaneous movements will generate motion artifacts around the organ of interest.

One way to reduce scan time is to employ a high-speed acquisition sequence to acquire under-sampled MRI and then improve its quality by postprocessing. Researchers have presented several conventional methods to restore under-sampled MRI images to achieve high-quality images. These methods, which include linear regression [6], compressed sensing [7, 8], and random forest [9, 10], can provide high quality MR images with short time. These conventional techniques can generate MR images with a high signal-to-noise ratio and a short scan time. However, the influence of extracting hand-crafted features and the difficulty of modulating parameters to optimize performance limit their utility for routine clinical applications [11].

An alternative method to obtain high-quality MR images from low-quality MR images is to apply a deep learning approach. Several researchers have used generative adversarial network (GAN) to produce high-quality MR images by inputting low-quality MR images [12, 13]. The task of improving the quality of under-sampled MR images using U-net was also reported [11, 14]. These studies provide several potential solutions to solve the problem of long MRI scan time for daily MR-linac applications [11–14]. However, applying deep learning in high-quality image generation is often challenged by limited training data, since it can be expensive and time-consuming to acquire enough paired-data of real-world patients in MR-linac.

In this paper, we proposed a deep learning method that could steadily improve image quality for accelerated 3D imaging. To address the difficulty of collecting patient data on MR-linac, we proposed a data augmentation technique to generate pseudo-linac MRI from simulation MRI for model pre-training. To the best of our knowledge, this is the first attempt to restore the image quality for high-speed scanning for the MR-linac. Different from most of current research, we used the real MR images, not the simulated images, to train the deep learning model. The results indicated that our method could achieve higher quality image compared with high-speed MR scanning and save 66% of the total generation time compared with low-speed MR scanning. The proposed method also can improve the accuracy of image registration for radiotherapy.

## Methods

### Data acquisition

Daily-MRI data were collected from 19 patients with prostate cancer who received MRI based adaptive radiotherapy (ART) from May 2021 to July 2022. Daily MRI scanning was performed on Unity MR-linac (Elekta, Stockholm, Sweden) using T2 3D LSHQ and T2 3D HSLQ sequences within 30 min, respectively. Sixty pairs of 1.5 T MR images in total from nineteen patients who received ART were retrospectively analyzed in this study. The number of collected paired image sets (LSHQ and HSLQ images) varied from 2 to 5 pairs for each patient. Imaging data of LSHQ and HSLQ images were 20,400 slices, respectively, for model training. The acquisition time of HSLQ (T2 3D Tra 2 min) and LSHQ (T2 3D Tra) sequence were 117 s and 411 s respectively. The MRI imaging protocols of HSLQ and LSHQ were shown in Additional file 1: Appendix A.

Furthermore, Images of 139 patients with prostate cancer were collected to train a data augmentation model to increase the size and diversity of the training set. Twenty of them, who were not included in the training of main model (19 patients), had 3.0 T simulation MR, LSHQ MR, and HSLQ MR images (2D slices: n = 6800). Simulation-MRI scanning was performed using a 3.0 T MRI simulator (Discovery MR750w, GE Healthcare) with a T2-fs-propeller sequence. The Simulation-MRI imaging protocols were shown in Additional file 1: Appendix A.

Institutional Review Board approval was obtained for this retrospective analysis, and the requirement to obtain informed consent was waived. All the patient data were de-identified.

### Data augmentation and training

**Fig. 1:**
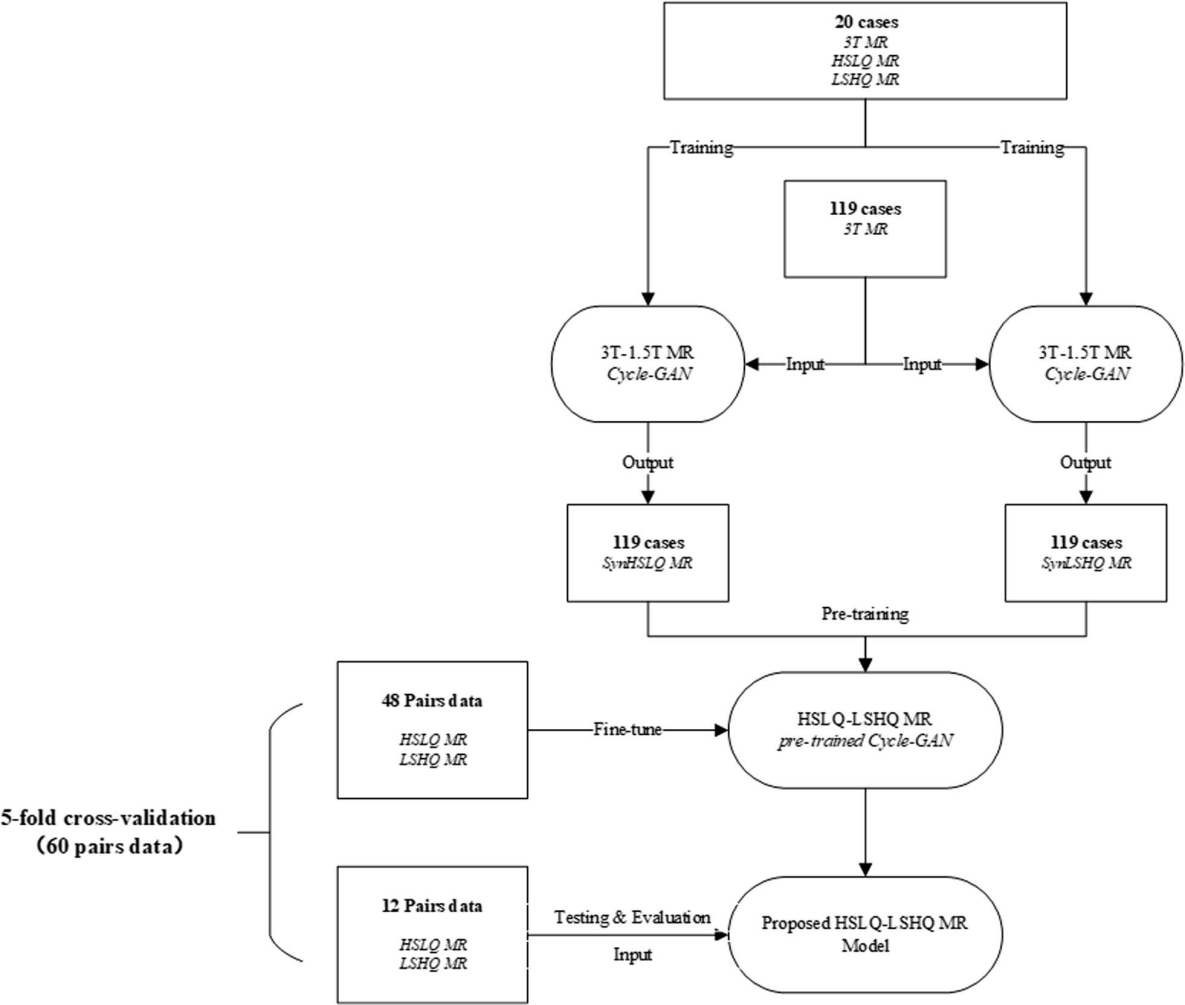
The workflow of data-augmentation and the training

To train an effective model, deep learning-based data-augmentation was applied to increase the size and diversity of the training set. The workflow of data-augmentation and the training step were shown in Fig. 1. Two cycle-GAN models, which was trained by twenty pairs of 3.0 T simulation MR, LSHQ MR, and HSLQ MR images, that can transform 3.0 T simulation MR images to synthetic HSLQ or synthetic LSHQ MR images were trained. Secondly, 3.0 T simulation MR images of the remaining 119 patients were enrolled into two cycle-GAN models to generate 119 sets of synthetic HSLQ and LSHQ MR images. Subsequently, a pre-trained model for high-resolution MR image generation was trained with these paired synthetic MR images.

To restore the image quality of high-speed scanning for the MR-linac, we used 60 pairs (from 19 patients) of daily MR images, including LSHQ and HSLQ MR images, to fine-tune the pre-trained model.

### Deep learning framework

**Fig. 2:**
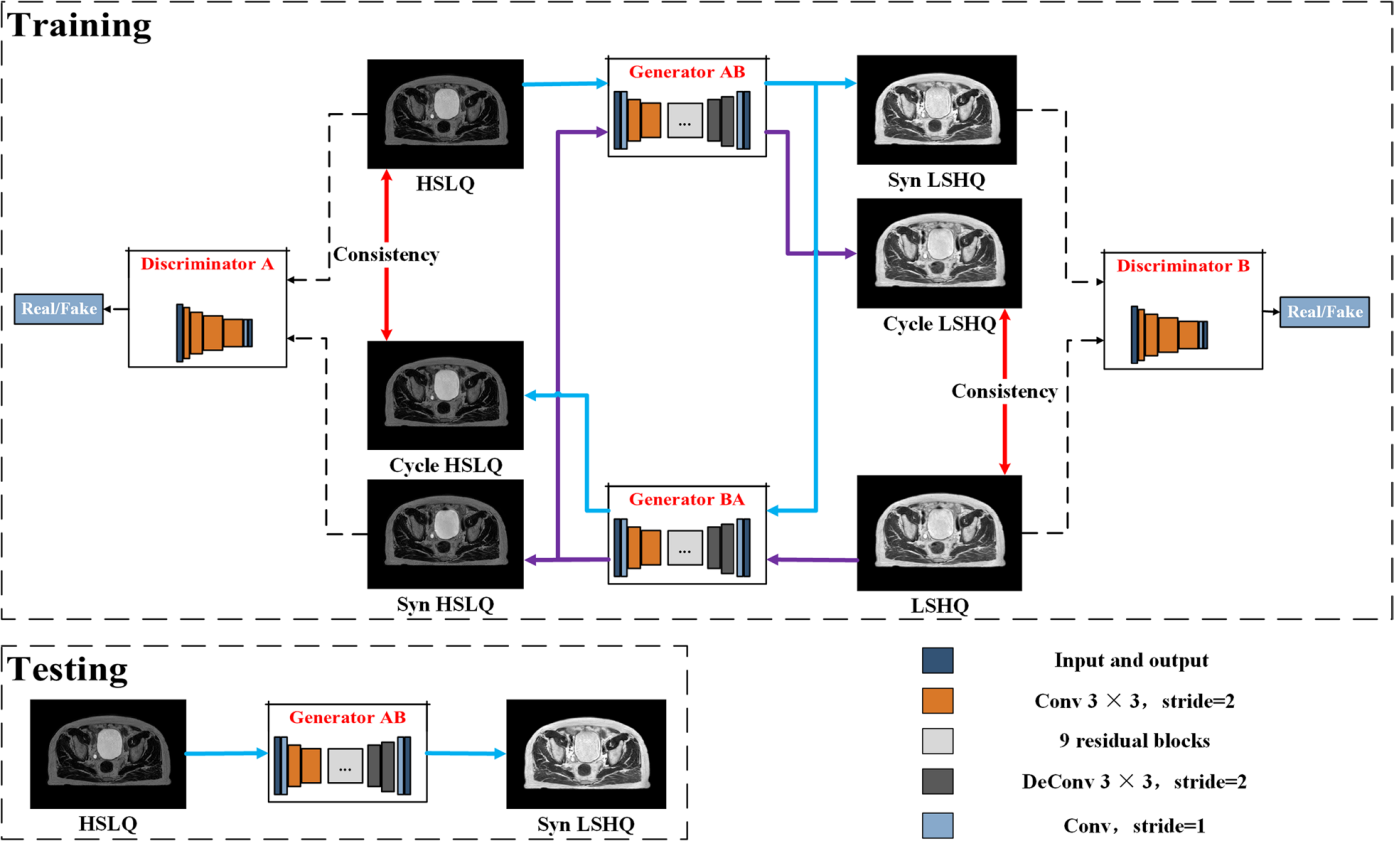
Illustration of the training and testing part of the CycleGAN framework

Figure 2 shows the workflow of the training and testing stages in the proposed framework. We employed a CycleGAN which consisted of two discriminators (D) and two generators (G) to learn the mapping between the HSLQ and LSHQ images [15]. When testing the trained model, synthetic LSHQ images were predicted by the Generator AB of CycleGAN with HSLQ images as the input [16]. The CycleGAN model is expected to obtain high-quality synthetic LSHQ (synLSHQ) images from the HSLQ images with high acquisition speed. The details of the generator and discriminator are shown in Additional file 1: Appendix B.

### Experiment

The five-fold cross-validation technique was employed to evaluate the proposed method. Here the data set (60 pairs of daily MR images) was split into 5 folds for the cross-validation [17]. Data for the same patient were selected into the same fold. During the first iteration, the first four folds (2D slices: 16,320) were used to fine-tune the model and the remaining one-fold (2D slices: 4080) was used to test the model. This process was repeated until each of the 5 folds was enrolled as the test set. In this experiment, model training and testing were done on an Nvidia GeForce RTX 3090 GPU.

### Evaluation

The benefit of the proposed synLSHQ, which was generated by our model, was analyzed in image quality, and registration accuracy.

#### Efficiency

The acquisition time of HSLQ, LSHQ and synLSHQ generation time is recorded so as to evaluate the efficiency of our proposed method.

#### Image quality

HSLQ and the proposed synLSHQ images were rigidly registered to the reference ones before the evaluation of image quality. The LSHQ images were set as the reference to evaluate the quality of HSLQ and the proposed synLSHQ images. The indices included the normalized mean absolute error (nMAE) [18, 19], structural similarity index measure (SSIM) [20], peak signal-to-noise ratio (PSNR) [21], and edge keeping index (EKI) [22]. Lower nMAE, larger SSIM, greater PSNR, and higher EKI values indicated better quality synthetic images. The definition of these evaluation indexes is presented in Additional file 1: Appendix C.

#### Registration accuracy

**Fig. 3:**
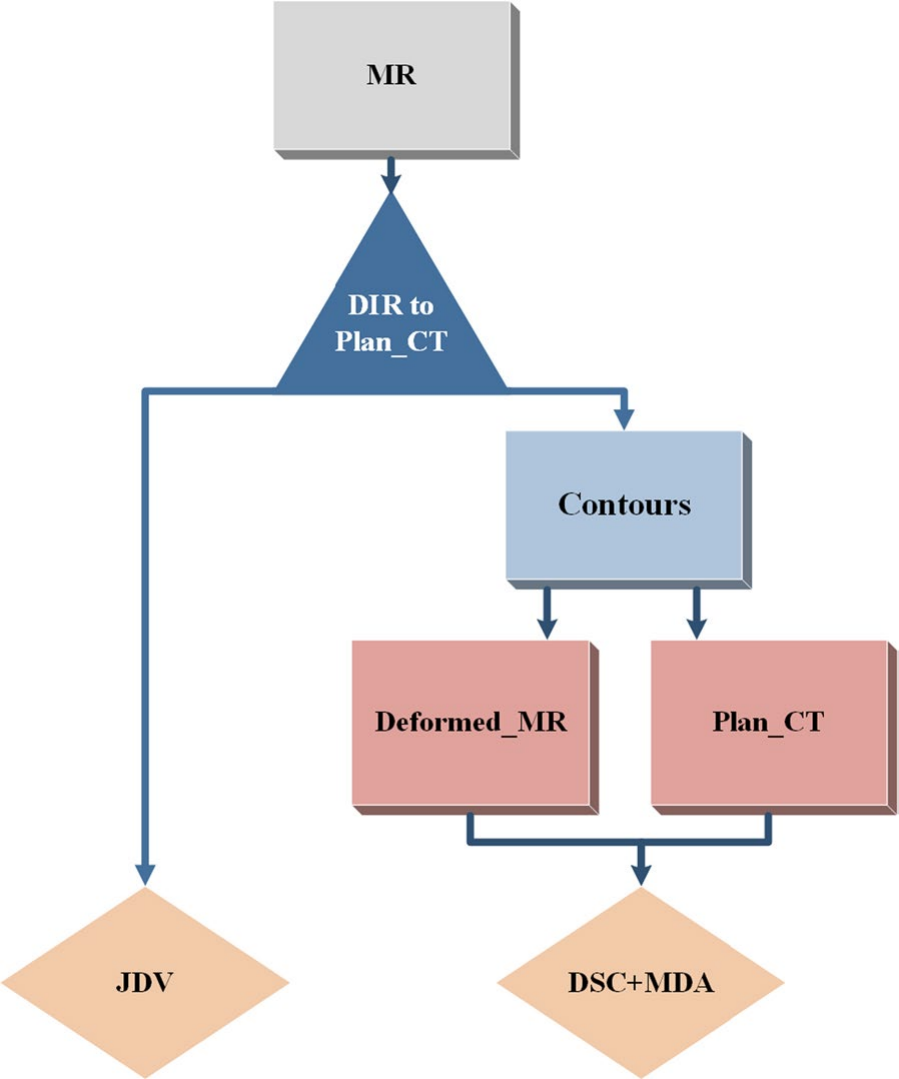
Workflow of Registration analysis. Evaluation metrics of registration: Jacobian determinant value (JDV); Dice similarity coefficient (DSC); Mean distance to agreement (MDA)

The daily LSHQ images were deformable registered against the planning CT to perform online adaptive radiotherapy process in clinic. Therefore, we evaluated the effect of image quality on deformable registration accuracy. The workflow of registration analysis is shown in Fig. 3. The synLSHQ and HSLQ images were deformably registered to the planning CT images, respectively. Jacobian determinant value (JDV) and geometric indices, which are two methods recommended by the AAPM Task Group 132 [23, 24], were used to analyze the registration accuracy. The average JDV close to 1 indicates a better registration [24]. The CTV and OARs were manually contoured on the planning CT, deformed HSLQ, and synLSHQ images by an experienced clinician. The contours were reviewed by two senior clinicians. The consistency of the corresponding region of interest on planning CT and MR images can reflect registration accuracy. The geometric indices included the Dice similarity coefficient (DSC) and mean distance to agreement (MDA). A higher DSC and lower MDA indicate better image registration.

## Results

Here, the performances of the synLSHQ images generated by the proposed method are analyzed by efficiency, image quality, and registration accuracy.

### Efficiency

The total averaged generation time (s/case) of our proposed method included HSLQ acquisition time (approximately 117 s) and synLSHQ generation time (approximately 22.6 s). Compared with the existing LSHQ acquisition approach that generally requires 411 s/case, our highly efficient model (total generation time: 139.6 s) yields greater promise to generate synLSHQ images for routine clinical use. Our proposed method (including HSLQ and synLSHQ generation time) saved a total time of up to 66% compared with the LSHQ scanning approach.

### Image quality

The results of image quality analysis between HSLQ and synLSHQ against the LSHQ images are shown in Table 1. The synLSHQ images generated by our method exhibited superior image quality with nMAE dropping by 57%, SSIM rising by 3.4%, PSNR increasing by 26.9%, EKI ascending by 3.6% compared with the HSLQ images, respectively. The statistical analysis in Table 1 further indicates that the image quality of the synLSHQ images was improved significantly compared with that of HSLQ (*p* < 0.01 for nMAE, SSIM, PSNR, and EKI).

**Table 1:** Quantitative image quality analysis between synLSHQ and HSLQ versus LSHQ images

	nMAE	SSIM		PSNR (dBs)	EKI
	Mean ± sd	Mean ± sd		Mean ± sd	Mean ± sd
HSLQ–LSHQ	0.14 ± 0.04	0.87 ± 0.02	HSLQ	20.95 ± 2.21	0.55 ± 0.06
synLSHQ–LSHQ	0.06 ± 0.01	0.90 ± 0.02	synLSHQ	26.59 ± 1.72	0.57 ± 0.06
synLSHQ versus HSLQ	*p* < 0.01	*p* < 0.01		*p* < 0.01	*p* < 0.01

*LSHQ*, LSHQ images; *HSLQ*, HSLQ images; *synLSHQ*, synthetic LSHQ images; *nMAE*, normalized mean absolute error; *SSIM*, structural similarity index measurement; *PSNR*, peak signal-to-noise ratio; *EKI*, edge keeping index

**Fig. 4:**
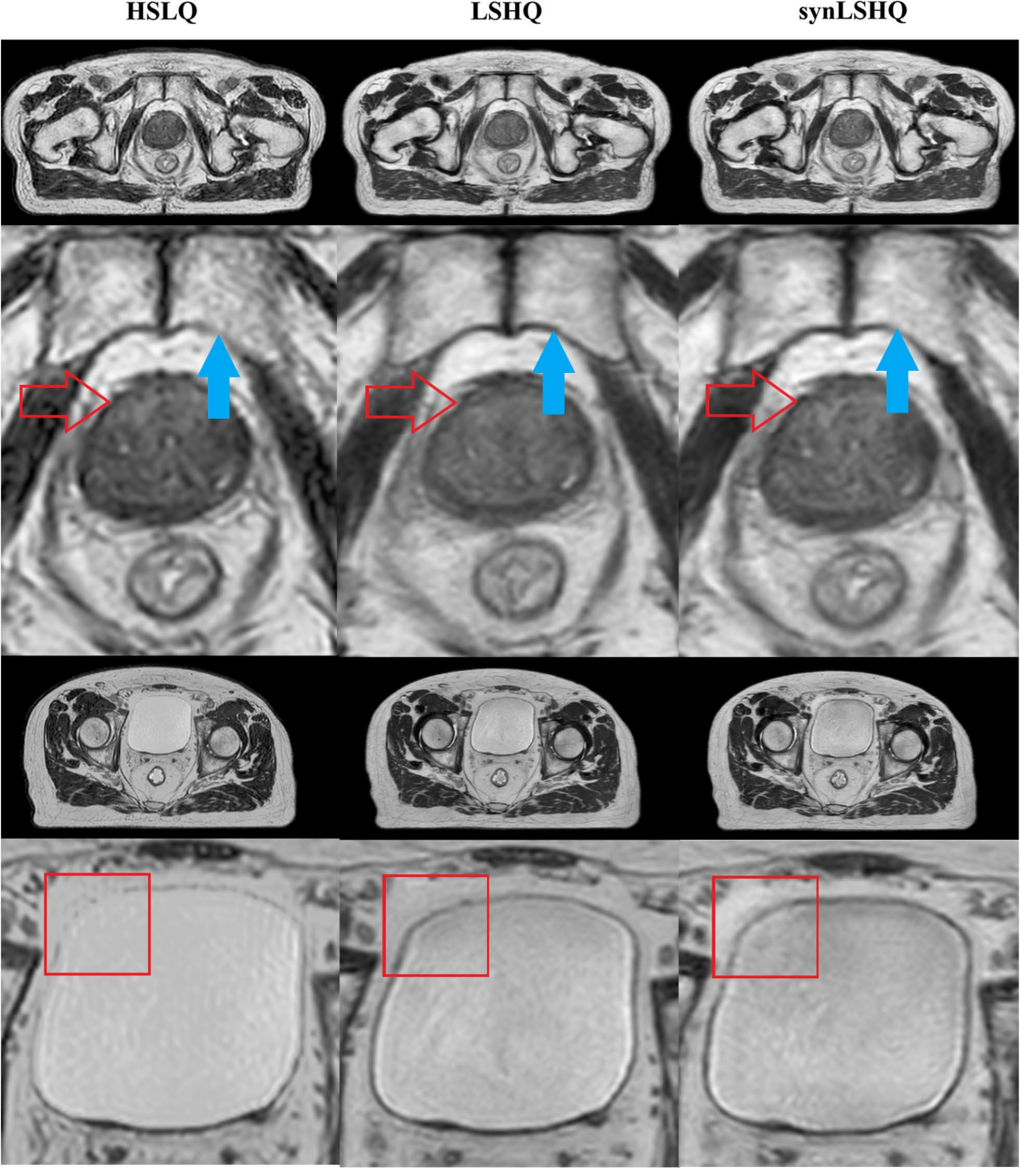
Examples of HSLQ, LSHQ, and synLSHQ images. First column: HSLQ; Second column: LSHQ; Third column: synLSHQ. Red arrow: boundary of the prostate; Sky blue arrow: boundary of the pubic symphysis; Red box: boundary of bladder

Figure 4 shows examples and corresponding zoom-in views of HSLQ, LSHQ, and the generated synLSHQ images. The zoom-in areas include the bladder and boundary of the pubic symphysis and prostate. The first two rows in Fig. 4 show that the boundary and soft tissue around the pubic symphysis and prostate in the synLSHQ image were easier to distinguish compared with that in the HSLQ images. The sky-blue arrow in the synLSHQ images shows a clear boundary and lower noise around the pubic symphysis. The synLSHQ images also provide a favorable image quality around the prostate (red arrow) compared with the HSLQ images.

The third and fourth row in Fig. 4 show that the streak artifacts appearing around the boundary of bladder (Red box) in the HSLQ images were corrected in the synLSHQ images. The synLSHQ images were more consistent with the LSHQ images.

### Registration accuracy

The Jacobian determinant and geometric indices (DSC and MDA) were performed to analyze the registration accuracy for each transformation. When deformed to reference planning CT images, the JDVs for the HSLQ and synLSHQ images were 0.83 ± 0.07 and 0.88 ± 0.04, respectively. The *p*-value was less than 0.01 and the synLSHQ images exhibited a higher mean JDV by 0.05 compared with the HSLQ images. These results indicate that deformed synLSHQ images experience less expansion and shrinking compared with deformed HSLQ images.

**Fig. 5:**
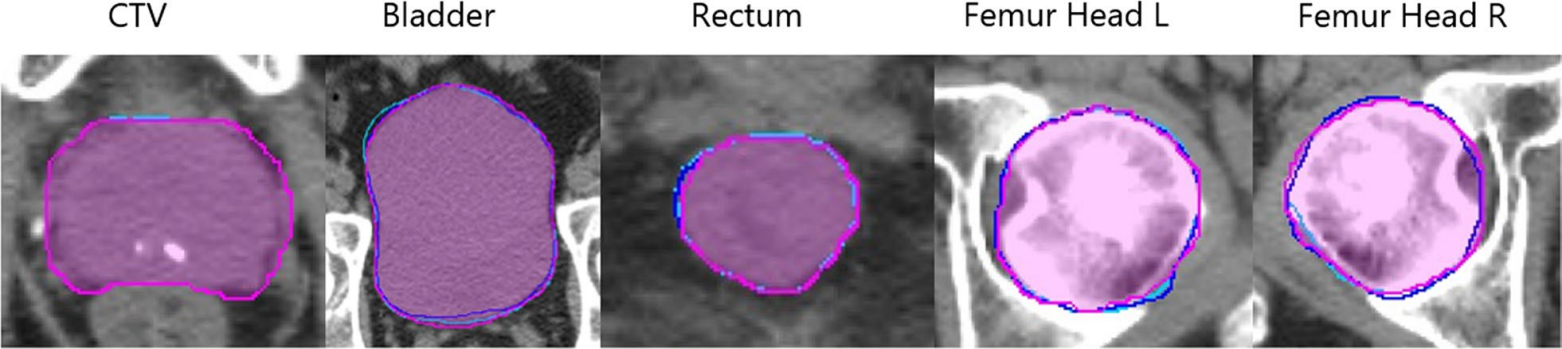
Examples of contours transferred to reference planning CT images. Purple colorwash: contours on planning CT image; Dark Blue line: contours on T2 HSLQ image; Sky blue line: contours on the T2 synLSHQ image

**Fig. 6:**
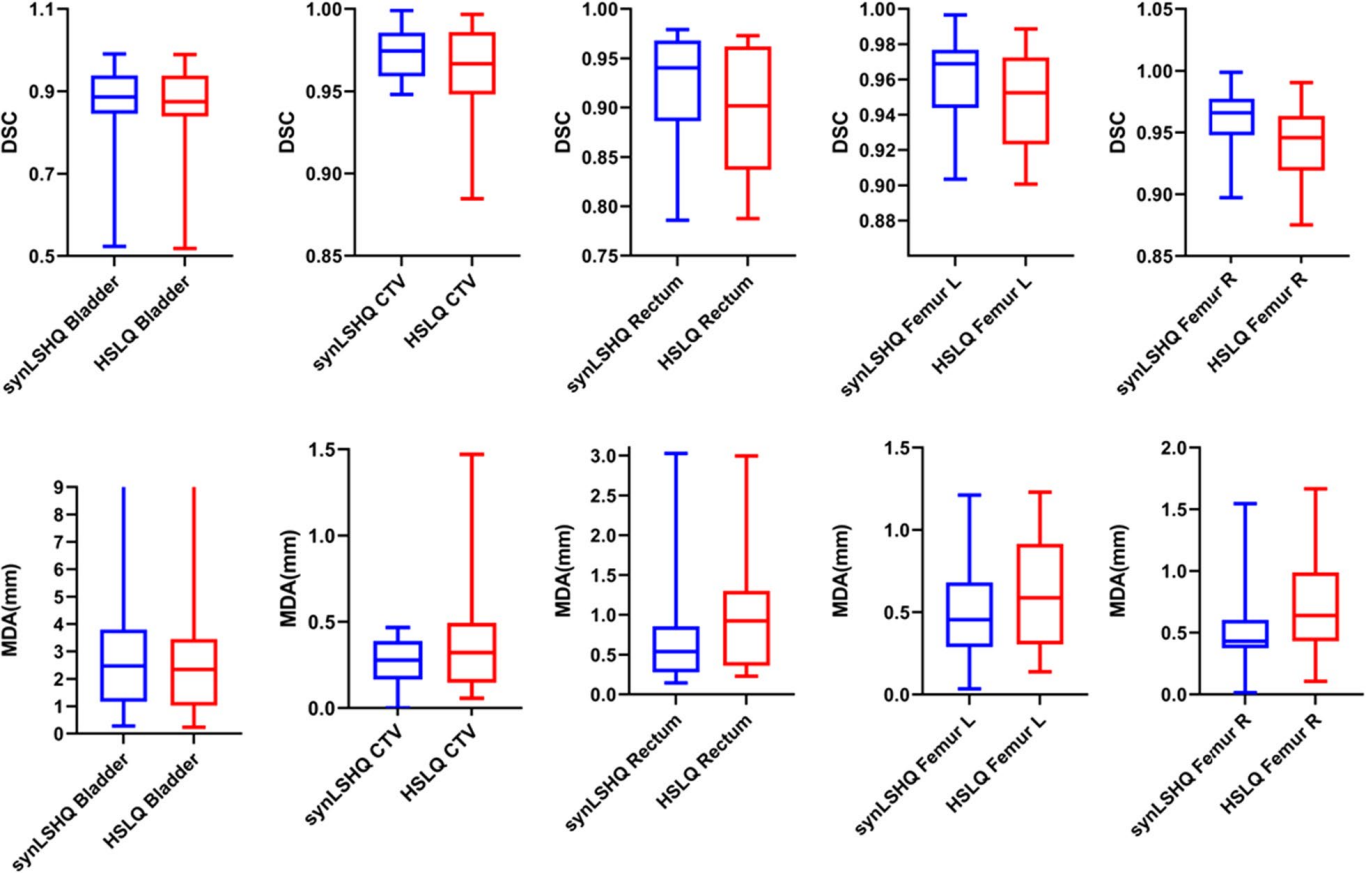
DSC and MDA box plots for the manual contours on the T2 HSLQ and synLSHQ images with respect to the reference planning CT images

Examples of the CTV, bladder, rectum, left femur head, and right femur head are shown in Fig. 5. The results of contours from the synLSHQ images are significantly close to that of the reference planning CT images. The DSC and MDA results of OARs for each transformation are shown in Fig. 6. On average, the synLSHQ images improved the DSC of the CTV, rectum, left femur head, and right femur head by 0.01 (*p* > 0.05), 0.03 (*p* < 0.05), 0.01 (*p* < 0.05), and 0.02 (*p* < 0.05), respectively, and reduced the MDA of the CTV, rectum, left femur head, and right femur head by 0.13 mm (*p* > 0.05), 0.24 mm (*p* < 0.05), 0.11 mm (*p* < 0.05), and 0.22 mm (*p* < 0.05), respectively, compared with the HSLQ images. Meanwhile, the equivalent contours error of the bladder (*p* > 0.05 for both DSC and MDA) were obtained from the HSLQ and synLSHQ images. These results demonstrate that the volumetric overlap ratio of the synLSHQ and planning CT images was higher compared with that of the HSLQ and planning CT images. The Jacobian determinant and the geometric indices demonstrate that the proposed synLSHQ can improve the registration accuracy compared with HSLQ images.

## Discussion

MRI guided adaptive radiotherapy is a useful technique; however, its time-consuming nature presents a significant challenge. The long period of adaptive treatment often affects patient comfort—especially patients with diminished bladder capacity. Moreover, long scan times also introduce motion artefact in MR imaging. These limitations impede the application of MRI guided adaptive radiotherapy. To improve patients’ comfort and reduce motion artefact, it is important to decrease the duration of each step of adaptive radiotherapy. The proposed synLSHQ has the potential to effectively address this challenge. In one case, our proposed method saved 66% of the total generation time. With process optimization and hardware development, this method has the potential to further improve efficiency to reduce high-quality MRI acquisition time.

There was improvement in nMAE (57%), SSIM (3.4%), PSNR (26.9%), and EKI (3.6%) compared with the HSLQ images. Overall, synLSHQ generated by the proposed method enhances the registration accuracy with a superior mean JDV (6%) and preferable geometric indices.

Several studies have demonstrated the feasibility of deep learning methods for synthesizing high-resolution MR images from low-resolution MR images [8, 12, 25]. Most of the paired data for training and validation was generated using down-sampling post-processing method in previous studies. In our study, we proposed a deep leaning-based data augmentation technique to overcome the problem of limited size and diversity of training data in clinic. And then we proposed an effective fine-tune method by using real paired data from the MR-linac to generate high-quality synthetic MRI images.

The proposed method showed potential for MRI guided adaptive treatment of prostate cancer. For the application, the first step is to obtain under-sampled MRI images using the HSLQ sequence. Then the synLSHQ images are generated through the proposed model by inputting HSLQ images. Finally, synLSHQ images are registered to planning CT images to determine whether to perform online adaptive radiotherapy process. As shown in the above application scenarios, the proposed method can significantly reduce scan time while ensuring image quality compared with LSHQ sequence.

Several limitations should be considered in our study. Because of lack of enough real-world data, an augmentation technique was used in training of the proposed model. There is no fully real-world data-based model compared with the proposed model. Fully real-world data-based model is meaningful for generating high quality MR in short time for precision radiotherapy in future work. The proposed method was tested with data collected in our department. External validation will be required before implementing our proposed model on other MR-linac machines. Meanwhile, our proposed model was trained for prostate cancer. Other models which are trained using data from other types of cancer should be carefully evaluated before clinical use.

## Conclusion

In conclusion, we proposed a novel method to improve image quality and registration accuracy for accelerated 3D imaging with a 1.5 T MRI radiotherapy system. It generates high-quality MRI images from HSLQ scanning sequences to significantly shorten the scan time while ensuring the accuracy of radiotherapy.

## Supplementary Information

**Additional file 1**. Appendix.

## Data Availability

The raw data supporting the conclusions of this article will be made available by the authors, without undue reservation.

## Abbreviations

MRI: Magnetic resonance imaging
GAN: Generative adversarial network
CycleGAN: Cycle-consistent GAN
LSHQ: Low-speed, high-quality
HSLQ: High-speed, low-quality
synLSHQ: Synthetic LSHQ
TR: Repetition time
TE: Echo time
FOV: Field of view
NAS: Number of signal averaged
nMAE: Normalized mean absolute error
PSNR: Peak signal-to-noise ratio
SSIM: Structural similarity index measurement
EKI: Edge keeping index
CTV: Clinical target volume
OARs: Organs at risk
JDV: Jacobian determinant value
DSC: Dice similarity coefficient
MDA: Mean distance to agreement
