# Supplementary material for: Improving accelerated 3D imaging in MRI-guided radiotherapy for prostate cancer using a deep learning method
Source: Radiat Oncol. 2023 Jul 1;18:108. doi: 10.1186/s13014-023-02306-4 (PMC10314402; doi:10.1186/s13014-023-02306-4)
Supplement: Supplementary file 1 — Additional file 1. Appendix. [file 13014_2023_2306_MOESM1_ESM.docx]

**Supplementary Material**

**Appendix A:** **Image acquisition information**

**Table 1.** MRI imaging protocol of HSLQ, LSHQ and 3.0T simulation-MRI images.

|  | HSLQ | LSHQ | Simulation-MRI |
| --- | --- | --- | --- |
| TE (ms) | 278 | 82 | 84.3 |
| TR (ms) | 1535 | 1300 | 13852 |
| Flip angle | 90° | 90° | 110° |
| Pixel spacing (mm) | 0.83 | 0.52 | 0.78 |
| Slice thickness (mm) | 1 | 0.6 | 3 |
| FOV (cm) | 40 × 40 × 30 | 40 × 44.8 × 25 | 40 × 40 × 40 |
| NEX | 2 | 2 | 2.5 |
| Matrix | 480 × 480 | 864 × 864 | 512 × 512 |

**Appendix B:** **The details of the generator and discriminator**


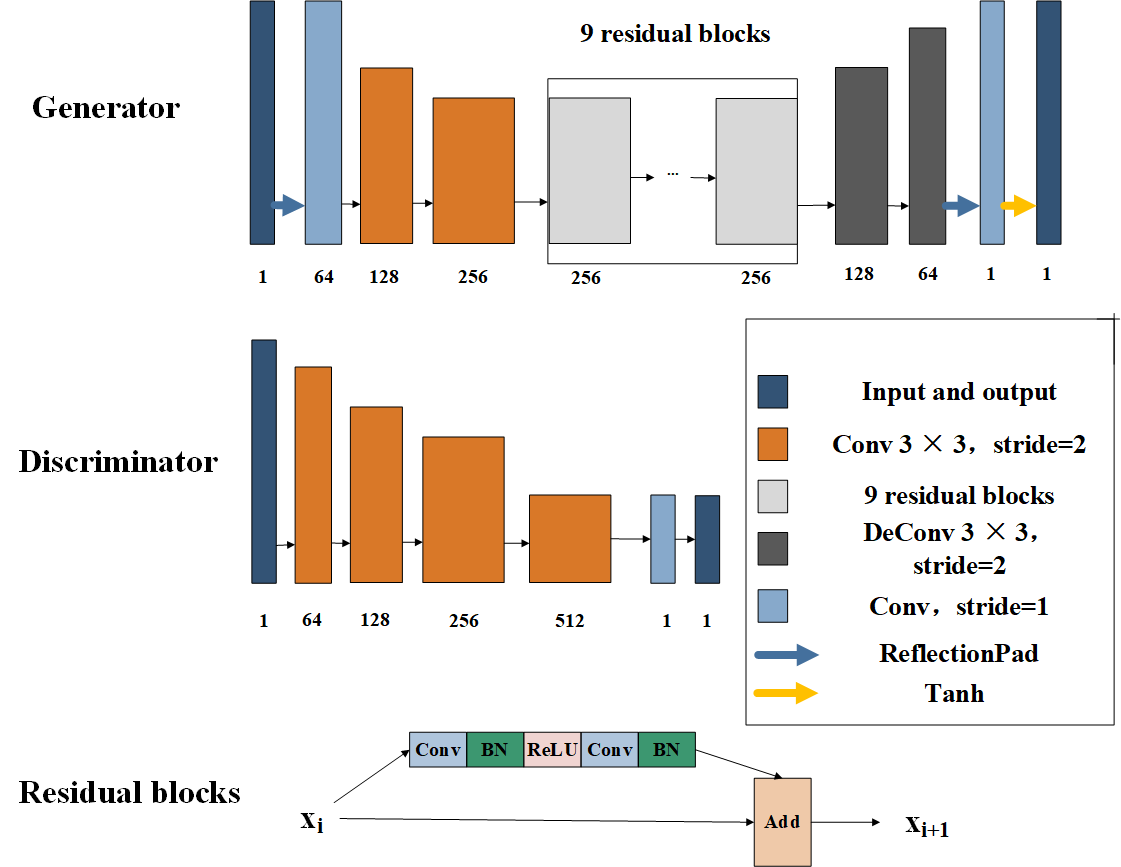


**Figure B.1.** Details of the CycleGAN network. Generator and discriminator in CycleGAN are shown on the first and second row. The third-row shows the architecture of the residual block which was used in the Generator. The numbers marked under each network block indicate the number of channels. Conv: convolutional layer; DeConv: transpose convolutional layer; BN: batch normalization layer; ReLU: rectified linear unit; Tanh is an activation function.

Figure B.1. shows the details of the generator and discriminator. Nine residual blocks between several down sampling and up sampling operators were used in the generator. The 3 × 3 convolutional layers and transposed convolutional layers were used for down sampling and up sampling. Reflection padding was done because the padding of the convolutional layers with a kernel size of 7 × 7 was 0, whereas that of the other convolutional layers was 1. Before the generation of the output, Tanh was used as an activation function. The residual block used a skip connection. The discriminator was a 70 × 70 Patch GAN, in which a pixel in the final feature map matrix corresponded to 70 × 70 pixels of the original image.

The loss functions were as follows:

The generator GAB was employed to translate style B into A as close as possible, and the discriminator DB distinguished the synthetic B from the actual B, which constituted an adversarial loss:

$\mathcal{L}_{\mathrm{GAN}}\left( G,D_{B},A,B \right)=\mathbb{E}_{B}\left[ \log D_{B}\left( X_{B} \right) \right]+\mathbb{E}_{A}\left[ \log{(1-D}_{B}\left( G_{\mathrm{AB}}(X_{A}) \right) \right]$ (B1)

Then, the synthetic B was translated into the cycle A by GBA, where the cycle-consistent loss was employed to maintain the image structure of A. For the two cycles:

$\mathcal{L}_{\mathrm{cyc}}\left( G,A,B \right)$ = $\mathbb{E}_{A}\left( G_{\mathrm{BA}}\left( G_{\mathrm{AB}}\left( X_{A} \right) \right), X_{A} \right)+ \mathbb{E}_{B}\left( G_{\mathrm{AB}}\left( G_{\mathrm{BA}}\left( X_{B} \right) \right), X_{B} \right)$ (B2)

We also employed the identity loss, which made the synthetic B generated from B by $G_{\mathrm{AB}}$ close to B:

$\mathcal{L}_{\mathrm{identity}}\left( G,A,B \right)$ = $\mathbb{E}_{A}\left( G_{\mathrm{BA}}\left( X_{A} \right), X_{A} \right)+ \mathbb{E}_{B}\left( G_{\mathrm{AB}}\left( X_{B} \right), X_{B} \right)$ (B3)

Therefore, the full loss function is:

$\mathcal{L}_{\mathrm{CycleGAN}}= \mathcal{L}_{\mathrm{GAN}}\left( G,D_{B},A,B \right)+ \mathcal{L}_{\mathrm{GAN}}\left( G,D_{A},A,B \right)+ {\lambda_{1}\mathcal{L}}_{\mathrm{cyc}}\left( G,A,B \right)+{\lambda_{2}\mathcal{L}}_{\mathrm{identity}}\left( G,A,B \right)$ (B4)

**Appendix C: Definition of indexes for image quality evaluation**

The image quality indices included the normalized mean absolute error (nMAE), structural similarity index measure (SSIM), peak signal-to-noise ratio (PSNR), and Edge keeping index (EKI). A lower nMAE, larger SSIM, greater PSNR and higher EKI value indicated better proposed synthetic images.

The MAE is the average value of the absolute error between the predicted value and true value. A lower MAE indicates a smaller error of the predicted value and is defined as:

$\mathrm{MAE}\left( g,f \right)=\frac{1}{N}\sum_{i=1}^{N} |g\left( i \right)-f\left( i \right)|$ (C1)

where g and f are the synthetic image and the reference image with N number of total pixels, respectively.

SSIM indicates the similarity between the reference and synthetic images. The range of SSIM is [0,1] where a larger value indicates less image distortion. SSIM is defined as:

$SSIM (g,f)=\frac{(2m_{g}m_{f}+c_{1}) (2s_{\mathrm{gf}}+c_{2})}{(m_{g}^{2}+m_{f}^{2}+c_{1}) (s_{g}^{2}+s_{f}^{2}+c_{2})}$ (C2)

where $m_{g}$ and $s_{g}^{2}$ are the [average](https://en.wikipedia.org/wiki/Average) and [variance](https://en.wikipedia.org/wiki/Variance) of g, respectively; $m_{f}$ and $s_{f}^{2}$ are the [average](https://en.wikipedia.org/wiki/Average) and [variance](https://en.wikipedia.org/wiki/Variance) of f, respectively; $s_{\mathrm{gf}}$is the covariance of g and f; $c_{1}= (k_{1}L)^{2},k_{1}=0.01$, $c_{2}= (k_{2}L)^{2},k_{2}=0.03$, and $L$ is the range of pixel values with a value of 2^B^-1.

PSNR is used to describe the ratio of the maximum possible power of a signal to the destructive noise power that affects its representation accuracy. PSNR is usually expressed in units of logarithmic decibels (dBs). A larger PSNR value indicates better image quality as follows:

$PSNR=10\cdot\log_{10} (\frac{{\mathrm{MAX}_{I}}^{2}}{\frac{1}{\mathrm{mn}}\sum_{i=0}^{m-1} \sum_{j=0}^{n-1} {[I\left( i,j \right)-K\left( i,j \right)]}^{2}})$ (C3)

where ${\mathrm{MAX}_{I}}^{2}$ represents the possible maximum pixel value of an image and m and n describe the size of the image.

Edge keeping index (EKI): This parameter is used to assess the edge preservation capacity. It is calculated as:

$EKI (g,f)=\frac{\sum_{i=1}^{N} (\Delta g_{i}-{\Delta\mu}_{g}) (\Delta f_{i}-{\Delta\mu}_{f})}{\sqrt{\sum_{i=1}^{N} {(\Delta g_{i}-{\Delta\mu}_{g})}^{2} \sum_{i=1}^{N} {(\Delta f_{i}-{\Delta\mu}_{f})}^{2}}}$ (C4)

where $g_{i}$ and $f_{i}$ are the reference and synthetic images, respectively. $\Delta g_{i}$ and $\Delta f_{i}$ are found by filtering $g_{i}$ and $f_{i}$ through a high pass Laplacian filter with the mean value as $\mu_{g}$ and $\mu_{f}$, respectively.
